# Excess Opioid Medication and Variation in Prescribing Patterns Following Common Breast Plastic Surgeries

**DOI:** 10.1177/22925503231172789

**Published:** 2023-05-26

**Authors:** Osama A. Samargandi, Colton Boudreau, Kaleigh MacIssac, Connor McGuire, Rawan ElAbd, Adel Helmi, David Tang

**Affiliations:** 1 Division of Plastic Surgery, Department of Surgery, 37848 King Abdulaziz University, Jeddah, Saudi Arabia; 2 Division of Plastic Surgery, 3688 Dalhousie University, Halifax, NS, Canada; 3 Faculty of Medicine, 3688 Dalhousie University, Halifax, NS, Canada; 4 Division of Plastic and Reconstructive Surgery, 5620 McGill University, Montreal, QC, Canada; 5 Division of Plastic Surgery, 8166 University of British Columbia, Vancouver, BC, Canada

**Keywords:** opioid, breast surgery, mastopexy, breast augmentation, breast reduction, abuse, pain management, sein, opération, opioïde, plastique, gestion de la douleur

## Abstract

**Purpose:** Excess opioid prescribing has societal impacts including addiction, dependence, and misuse. This study aims to investigate prescribing patterns and self-reported patient experiences with opioid use, pain control, and disposal of unused medication following common breast surgeries. **Methods:** A total of 46 patients undergoing 5 breast procedures were identified during a predefined 14-week period. All procedures were carried out at a single tertiary care hospital by 9 plastic surgeons. Provincial narcotic monitoring program provided linked prescription information for identified patients. All patients were invited to participate in a telephone interview regarding postoperative opioid use. **Results:** A total of 41.6% of patients received and filled an opioid prescription following a breast procedure. Hydromorphone was the most commonly prescribed narcotic. The average number of opioid tablets dispensed following breast procedures was 31.9. Four percent of breast patients required an opioid refill. A total of 75% of breast patients used at least 1 over-the-counter analgesic, most commonly acetaminophen alone. Average self-reported pain score and total pain period were not significantly different between those using opioids and those not. A total of 6.7% and 23.1% of patients report returning excess narcotics to a pharmacy, while the majority report still having or self-disposing of excess tablets. **Conclusions:** Opioids are prescribed in excess for the breast procedures we analyzed. The majority of unused opioids were noted to still be at home or disposed of inappropriately. This suggests a role for reviewing opioid-prescribing patterns for common plastic surgery procedures to reduce the burden of the ongoing opioid epidemic.

## Résumé

**Objectif:** L’excès de prescriptions d’opioïdes a des conséquences sociétales, y compris l’addiction, la dépendance et le mésusage. La présente étude vise à examiner les profils de prescription et les expériences déclarées par les patients à l’égard de l’utilisation d’opioïdes, du contrôle de la douleur et de l’élimination de médicaments non utilisés après des opérations mammaires courantes. **Méthodologie:** Au total, 46 patientes ont été soumises à cinq interventions mammaires pendant une période prédéfinie de 14 semaines. Toutes les interventions ont été réalisées par neuf chirurgiens plastiques d’un même hôpital de soins tertiaires. Le programme provincial de surveillance des narcotiques a fourni de l’information liée aux prescriptions de ces patientes. Toutes les patientes ont été invitées à participer à une entrevue téléphonique au sujet de l’utilisation d’opioïdes en période postopératoire. **Résultats:** Au total, 41.6% des patientes ont reçu et utilisé une prescription d’opioïdes après une intervention mammaire. L’hydromorphone était le narcotique le plus prescrit. En moyenne, 3.9 comprimés d’opioïdes ont été administrés après les interventions mammaires. Quatre pour cent des patientes ont demandé un renouvellement de prescription. Ainsi, 75% des patientes ont utilisé au moins un analgésique en vente libre, généralement sous forme d’acétaminophène seule. Le score moyen de douleur autodéclaré et la période totale de la douleur n’étaient pas sensiblement différents entre ceux qui prenaient des opioïdes et ceux qui n’en prenaient pas. Au total, 6.7% et 23.1% des patientes déclarent avoir retourné les excès de narcotiques à la pharmacie, tandis que la majorité d’entre elles indiquent les avoir conservés ou les avoir jetés elles-mêmes. **Conclusions:** Des excès d’opioïdes ont été prescrits lors des interventions mammaires analysées. La majorité des opioïdes non utilisés étaient encore chez les patientes ou avaient été éliminés de manière inappropriée. Ces constats font ressortir l’intérêt d’examiner les profils de prescription d’opioïdes lors des chirurgies plastiques courantes afin de réduire le fardeau de l’épidémie continue d’opioïdes.

## Background

For many patients, the postoperative period serves as one of the common initials means to get an opioid prescription, and potentially, become a frequent user. If the patients reach adequate pain control following their surgery, patients are left with excess opioid tablets. This puts patients at risk of transitioning into long-term opioid users.^1^ Surgeons have been under the spotlight for prescribing opioids in excess, which could potentially be a source of diversion and abuse.^1,2^ Plastic surgeons are not innocent and contribute to the opioid epidemic.^2^ Several studies evaluating common plastic surgery procedures have observed a practice of opioid overprescribing.^3–5^ These studies have highlighted a pattern of patients receiving excess opioid medication after undergoing plastic surgery; however, to our knowledge, there have been few data to explicitly quantify the volume of excess opioid pills following common breast plastic surgeries. We believe that the data of this study can help guide plastic surgeons on typical opioid consumption following breast plastic surgery and thus predict the optimal requirement of opioid tablets.

The purpose of this study is to determine the total number of opioid tablets prescribed following common breast plastic surgeries and the number of tablets left unused. Additionally, we aim to examine prescribing patterns and self-reported patient experiences with opioid use, pain control and disposal of unused medication.

## Methods

### Patient List Generation

Five commonly performed breast plastic surgeries in our institution were identified, including insertion tissue expander, unilateral augmentation mammoplasty, bilateral augmentation mammoplasty, unilateral reduction mammoplasty, and bilateral reduction mammoplasty. A specific breakdown of the proportion of each procedure is described in Table 1. For each procedure, the Nova Scotia Medical Service Insurance billing code was obtained. A list of procedure names and billing codes was provided to the Nova Scotia Health Authority (NSHA) Health Information Services (HIS) and lists were generated for all patients undergoing the 5 breast procedures. A 3-month date range was specified (October 1, 2018, to January 1, 2019, inclusive) and only procedures performed within these dates were considered for inclusion. Additionally, the search performed by HIS was restricted to billings by 9 plastic surgeons from 1 academic tertiary care hospital (QE2 Health Science Centre, Halifax Infirmary) located in Halifax, Nova Scotia, Canada. The output list from HIS was securely transferred to the investigators and variables included patient name, procedure, procedure date, billing code, procedure laterality (if applicable), Nova Scotia health card number, and telephone number. Institutional ethics approval was obtained from NSHA Research Ethics Board (Reference No. 1023725).

**Table 1.** Distribution of Breast Plastic Procedure Types.

Breast plastic procedures (*N* = 46)	
Procedure	Percentage of total procedures
Bilateral reduction mammoplasty	47.9% (22)
Unilateral reduction mammoplasty	26.1% (12)
Bilateral augmentation mammoplasty	17.4% (8)
Insertion tissue expander	4.3% (2)
Unilateral augmentation mammoplasty	4.3% (2)

### Data Linkage

Ethical approval allowed the secure transfer of patient data to the Nova Scotia Provincial Monitoring Program (NSPMP), a program that monitors the prescribing of all opioid medications in Nova Scotia. Patient health card number, procedure billing code, and date of procedure were securely transferred to collaborating statisticians at NSPMP. The program only provides data on prescriptions that were dispensed at a pharmacy and returned a list of prescription information linked to each health card number submitted. Due to personal health information protection, only the procedure type and related prescription data were returned, as to prevent the linkage of prescription information to the patient name. From this data, opioid dispensing rates and details for dispensing by the individual procedure were obtained. Additionally, details of the medications and dosing were extracted from this data set.

### Telephone Interviews

**Figure 1.**
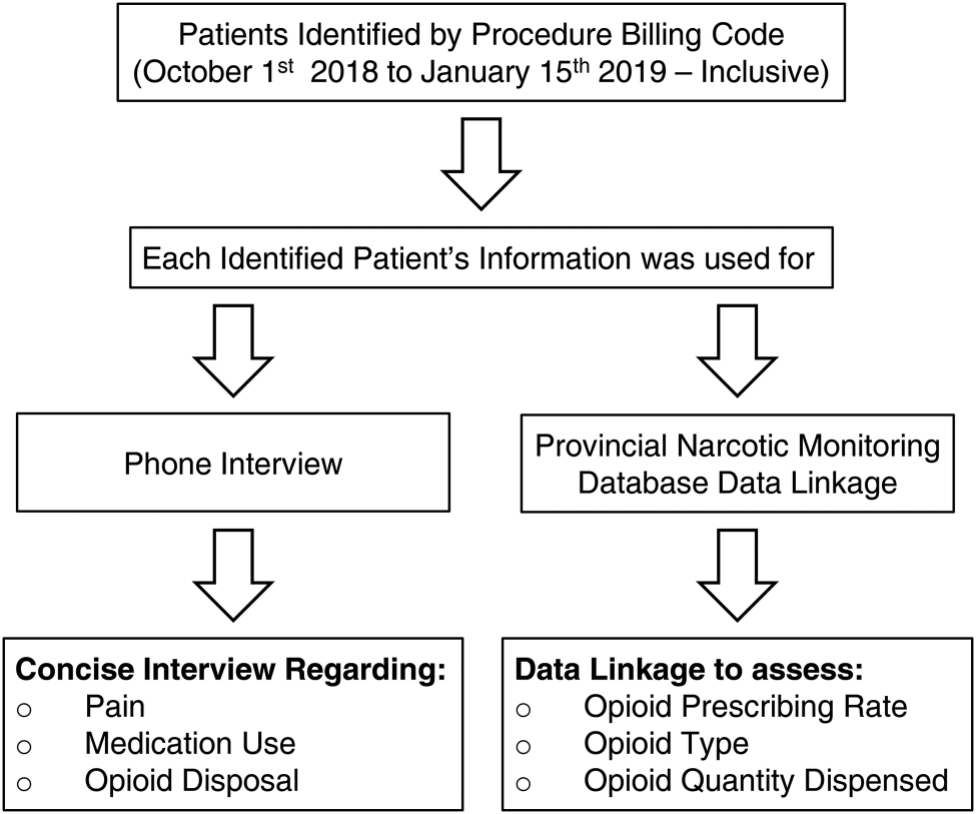
Summary of patients’ identification, data collection, and phone interview process.

Using patient lists generated by HIS, all potential participants identified were contacted using the telephone number provided. All participants were called up to 3 times, with a minimum of 5 rings each time. If the participant did not answer after 3 attempts, they were excluded from the self-reported interview data. Additionally, there were cases in which the telephone number was not current or was incorrect from the data received from HIS. In these cases, the participants were considered unreachable and excluded. See Figure 1 for more details.

**Figure 2.**
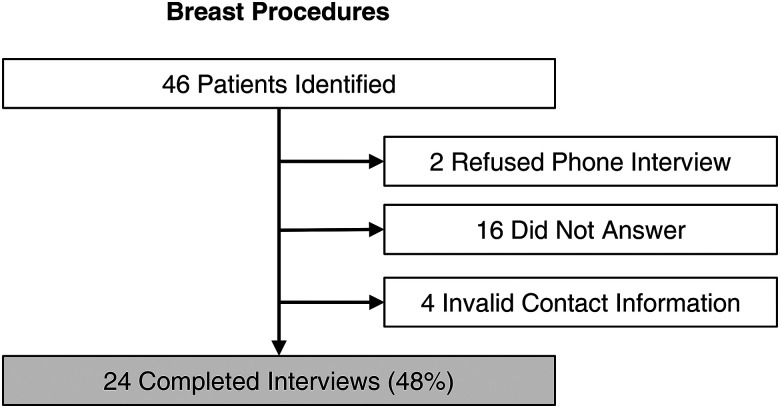
Flow diagram describes the number of patients included and details of phone interviews.

Each participant who was reached via telephone was first given a brief overview of the study and a verbal confidentiality notice as outlined in a standardized telephone script (Supplemental Document 1). Verbal consent to participate was then obtained. Participants who elected to not participate were excluded from the self-reported data set (Figure 2).

The standardized telephone script (Supplemental Document 1) was discussed with the participants. They were asked if they recalled receiving a prescription postoperatively and if so if they recalled details of any prescriptions such as medication name, dose or number of tablets. They were then asked about any over-the-counter (OTC) medications they may have used throughout the recovery period. The patients were also asked to estimate how many of the tablets of opioids they may have used, and any refills they may have required and then were asked for details about the disposal of any unused medications. The interviews elicited details on pain management postoperatively using the well-established McGill Pain Questionnaire format.^6^ Participants rate their pain from no pain (0) to the worst pain of their life (10). Inquiries were made regarding pain specifically in the first 24 h period, and then again in the 24-to-72-h period. Participants were then asked to estimate the total pain period, which was referred to as the length of time from the day of surgery until pain was no longer noticed.

### Statistical Analyses

Descriptive statistics were calculated using Microsoft Excel Version 16.16.3 (Microsoft®, Redmond, Washington). Figures were developed using Microsoft PowerPoint Version 16.16.3 (Microsoft®, Redmond, Washington). Tables were developed using Microsoft Word Version 16.16.3 (Microsoft®, Redmond, Washington). Average pain scores in Table 6 were calculated using student *t*-tests in Microsoft Excel, with *P* < .05 indicating significance.

## Results

During a 3-month period, a total of 46 patients who underwent breast plastic surgeries were identified. Of them, 24 completed interviews (52%). Figure 2 shows a detailed flow diagram.

### Database Results

**Figure 3.**
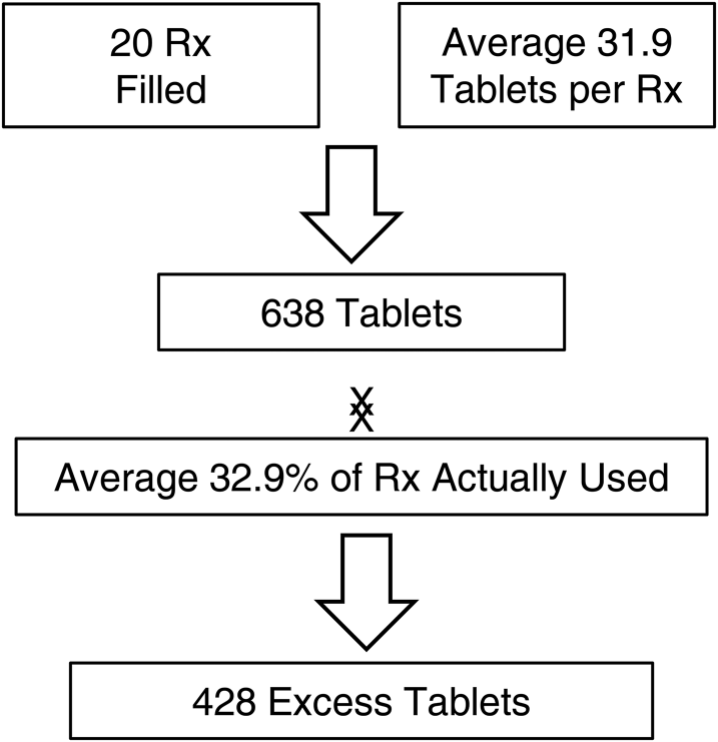
The total number of prescriptions filled for breast procedures.

The most commonly performed breast plastic surgeries were bilateral reduction mammoplasty (47.9%), unilateral reduction mammoplasty (26.1%), and bilateral augmentation mammoplasty (17.4%). Figure 3 details the overall number of prescriptions filled for breast procedures. For breast procedures, an average of 32.9% of prescriptions were used which lead to an excess of 428 tables over the 3-month catchment period. The overall dispensing rate of patients who received and filled an opioid prescription was 43.5% (Table 2). When looking at dispensing rates by procedure, for breast procedures, rates were highest for bilateral reduction mammoplasty (52.2%), bilateral augmentation mammoplasty (50%), and unilateral augmentation mammoplasty (50%). None of the patients who had undergone a tissue expander insertion procedure received an opioid prescription.

**Table 2.** Opioid Dispensing Rate of Various Breast Procedures.

	Breast procedures (*N* = 46)
Overall dispensing rate	
Percentage of patients who received and filled an opioid prescription	43.5% (20)
Percentage of patients who received and did not fill an opioid prescription	56.5% (26)
Dispensing rate by procedure	
*Breast procedures*	
Bilateral reduction mammoplasty	52.2% (12)
Bilateral augmentation mammoplasty	50.0% (4)
Unilateral augmentation mammoplasty	50.0% (1)
Unilateral reduction mammoplasty	23.1% (3)
Insertion tissue expander	0.0% (0)

Table 3 details the opioid prescription details for common breast plastic procedures. The most common type of opioids dispensed were hydromorphone 1 mg and 2 mg tablets were also the most common (45% and 45%; Table 3).

**Table 3.** Opioid Prescription Details for Common Breast Procedures.

Prescribed opioid and quantity of tablets for dispensed prescriptions		
Breast procedures		
Opioid (dose)	Proportion of total opioid prescriptions (*N* = 20)	Average number of tablets of opioid dispensed
Hydromorphone (1 mg)	45.0% (9)	16.4
Hydromorphone (2 mg)	45.0% (9)	35.4
Tylenol No. 3 (300 mg)	5.0% (1)	40.0
Ratio—Oxycocet (5/325 mg)	5.0% (1)	40.0
Average number of dispensed tablets for all breasts procedures		
Average number of tablets	31.9	

### Patient Interview Results

Self-reported prescription information is detailed in Table 4. Over half of the postoperative breast surgery (54.2%) patients recall receiving a postoperative opioid prescription while the majority did not require at least 1 refill (95.8%). A total of 75% of patients recall using at least 1 type of OTC analgesic, which most commonly included acetaminophen or a nonsteroidal antiinflammatory drug (NSAID; Table 4). Respondent self-reporting of OTC medication use is shown in Table 5. Of the involved patients, 75% of patients used 1 or more OTC medications during their recovery. The most commonly used OTC medication was acetaminophen (50.0%) and NSAIDs (22.2%). Acetaminophen and NSAIDs were commonly used in combination with 22.7% of respondents reporting this combination. One patient (5.6%) reported using medical cannabis following 1 breast surgery. For those patients reporting using at least 1 OTC medication, 88.9% of patients also reported using an opioid medication.

**Table 4.** Postoperative Self-reported Prescription Medication.

Self-reported prescription analgesia	
Percentage of interview respondents who recall receiving a postoperative prescription	Breast procedures (*N* = 24)
Yes, received	54.2% (13)
No, not received	41.6% (10)
Unsure	4.2% (1)

Self-reported prescription refill rates	
Percentage of interview respondents who recall requiring at least 1 refill on postoperative prescription	Breast procedures (*N* = 24)
Yes, refill received	4.2% (1)
No, refill not received	95.8% (23)
Unsure	0.0% (0)

Self-reported prescription detail awareness	
Percentage of interview respondents who recall details of the specific medication received	Breast procedures (*N* = 24)
Yes, aware of medication details	4.2% (1)
No, unaware of medication details	95.8% (23)

**Table 5.** OTC Analgesic Use in Postoperative Patients.

Self-reported postoperative over counter analgesics	
Percentage of interview respondents who recall using at least 1 OTC analgesic	Breast procedures (*N* = 24)
Yes, used at least 1 OTC analgesia	75.0% (18)
No, did not use OTC analgesia	25.0% (6)

Percentage of interview respondents using each type of OTC medication(s)	Breast procedures (*N* = 18)
Acetaminophen alone	50.0% (9)
NSAID alone	22.2% (4)
Acetaminophen + NSAID	22.2% (4)
Medical Cannabis	5.6% (1)

Percentage of interview respondents who recall using at OTC analgesics and opioid medication in	Breast procedures (*N* = 18)
Yes, used OTC medication and opioid	88.9% (16)
No, used only OTC medication	11.1% (2)

Abbreviations: NSAID, nonsteroidal antiinflammatory drug; OTC, over-the-counter.

Postoperative pain results indicate that there was no statistically significant difference in pain scores for patients who used or did not use opioids for pain control within the first 72 h (*P* > .05; Table 6). There was also no statistically significant difference in the self-reported postoperative total pain period in patients who used or did not use opioids (*P* > .05; Table 6).

**Table 6.** Self-Reported Postoperative Pain.

Self-reported postoperative pain using the Montreal pain questionnaire		
	Breast procedures (*N* = 24)	
Time period	<24 h	24–72 h
Respondents who used opioid	5.21 ± 1.44	5.44 ± 1.30
Respondents who did not use opioid	4.71 ± 1.87	4.44 ± 2.04
*P*-value	0.874	0.122

Self-reported postoperative total pain period (days)	Breast procedures (*N* = 24)
Respondents who used opioid	15.4 ± 6.2
Respondents who did not use opioid	16.8 ± 9.2
*P*-value (comparing opioid use vs no opioid in a specified period)	0.237

None of the patients used the entire opioid prescription (Table 7). In terms of disposal methods of unused opioids, the majority of patients still had the remaining tablets at home (61.5%) and only 23.1% used self-disposal. Only a small percentage of patients returned the unused pills to the pharmacy (7.7%; Table 7).

**Table 7.** Self-Reported Opioid Use and Disposal of Unused Medications.

Self-reported estimated opioid prescription use	Breast procedures (*N* = 13)
Estimated percentage of total opioid prescription used	32.9 ± 8.9
Percentage of patients reporting using the entire prescription	0.0% (0)

Self-reported disposal method of unused opioids	Percentage of respondents utilizing a method of disposal
Method of disposal	Breast procedures (*N* = 13)
Still have the remaining tablets at home	61.5% (8)
Returned to pharmacy	7.7% (1)
Self-disposal (toilet, garbage, sink)	23.1% (3)
Unknown	7.7% (1)

## Discussion

This study describes the prescribing patterns of common breast plastic surgeries postoperative opioid analgesia at a single tertiary care hospital, as well as the patient experience regarding pain management in the postoperative period. The results of this study show a noticeable variation in postoperative opioid-prescribing patterns and a tendency to overprescribe patients with opioid tablets. We identified that during 13 weeks, an excess of approximately 428 (out of 638; 67%) opioid pills were prescribed following 4 common breast surgeries at our institution. There were no refills of opioids required. The highest dispensing rate of postoperative opioid pills was observed among women who had undergone bilateral reduction mammoplasty. Hydromorphone was the most commonly prescribed opioid. The average number of dispensed tablets for all procedures was 32. The vast majority of patients did not properly dispose of the leftover pills.

Our results support the findings of Hart et al,^3^ as they reported that they achieved adequate pain control but with a substantial number of leftover pills following breast reduction and secondary breast reconstruction surgery. We agree that a prescription of 30 opioid tablets after breast plastic surgery is unnecessary and excessive. Similar results were also reported by Rose et al^5^ that plastic surgery patients are left with half of the amount of the needed opioid pills. The practice of overprescribing opioid pills in general has been attributed to several factors. Those include the lack of awareness among surgeons of how commonly patients are left with excess opioid pills, the concern that patients may have an unsatisfactory experience and the significant heterogeneity among plastic surgery patient populations and procedures.^1^ All the aforementioned factors make developing practice guidelines for postoperative opioid prescription practices a challenging task.

The opioid drug abuse epidemic led to the creation of federal and state government policies to control prescriptions. Such regulations differ by country and state but they are all in one way or another linked to prescription drug monitoring programs, electronic prescriptions, state initiatives, and continuing medical education requirements.^2,7^ In addition, the Food and Drug Administration oversees many of the policies and attempts to control opioid prescription practices by creating risk evaluation and mitigation strategies, which is a comprehensive training program on opioid use for prescribers, the effectiveness of which is still in doubt.^7^

Olds et al^8^ found that there is a significant risk of persistent opioid use after plastic and reconstructive procedures, with the greatest odds found in patients who underwent breast plastic surgery. Therefore, plastic surgeons should make serious efforts to minimize the need for opioids for pain control after surgeries. Such efforts include the use of multimodal pain management therapy, enhanced recovery after surgery (ERAS) protocols, and patient education and counselling to manage postoperative pain expectations. These ERAS protocols are just gaining acceptance in plastic surgery,^6,^^9–11^ and acknowledging the impact that multimodal analgesia can have on decreasing perioperative pain and opioid use after surgery is vital to its integration to practice. There is evidence in the literature to support the efficacy of perioperative nerve blocks^12,13^ such as the pectoralis muscle nerve block^14^ and paravertebral block,^15^ as well as the use of gabapentin,^16^ longer-acting local anesthetics, such as bupivacaine,^17^ and nonsteroidal antiinflammatory agents and cyclooxygenase- 2 inhibitors^18^ in limiting postoperative pain and the need for an opioid in different breast surgeries. For example, a randomized control trial by Parsa et al^18^ on 695 patients undergoing breast augmentation found that a single 400-mg dose of celecoxib administered 30 min before surgery significantly decreased opioid analgesic requirements after subpectoral breast augmentation when compared to placebo (*P* < .001). The authors recommended the administration of celecoxib 30 min before surgery to decrease opioid requirements after surgery. In another study, a multimodal approach that focuses on the administration of preoperative gabapentin and celecoxib in addition to the local anesthesia received during surgery allowed patients to have an opioid-free bilateral breast reduction and resulted in significantly less use of postoperative opioids, morbidity, and unplanned hospital admissions when compared to the group that underwent breast reduction under general anesthesia with the use of opioids, so-called the “traditional way.”^19^ In this study, a total of 75% of patients recall using at least 1 type of OTC analgesic, which most commonly included acetaminophen or an NSAID. Postoperative pain results indicate that there was no statistically significant difference in pain scores for patients who used or did not use opioids for pain control within the first 72 h.

Our study showed that more than half of patients still had the remaining tablets at home with no proper plan or awareness on how to properly dispose them. This problem is not uncommon among surgery patients and can potentially lead to the diversion of opioids for misuse or abuse.^1,3–5^ A previous study has found that only 5% of patients being prescribed opioids after surgery received proper disposal information.^20^ Excess opioid leftover pills can set the stage for improper storage; thus, stockpiling is an important contributor to their nonmedical use. A direct prescription from healthcare providers has been found to be the second largest source of misused opioids based on the 2020 American National Survey on Drug Use and Health.^9^ To facilitate the correct disposal of excess opioids by patients, preoperative education and counselling regarding proper use are essential. One way to mitigate this is to deliver proper information leaflets to patients perioperatively and include the use and misuse of opioids in the preoperative discussion.^2,20,21^ Other factors include onsite medication depository at the hospital.^21^

In summary, the current evidence in the literature supports the use of minimal preoperative fasting, opioid-sparing perioperative medications and use anesthetic techniques that decrease postoperative pain, as well as the use of measures to prevent intraoperative hypothermia and support early postoperative mobilization in an attempt to enhance the patient operative experience while maintaining safety.^11^ Surgeon education regarding the opioid epidemic as well as patient perioperative counselling on pain expectation, medication, and proper disposal of opioids play a huge role in preventing misuse. Notably, a previous study showed that implementing a mandatory educational intervention for opioid prescribers that includes evidence-based prescribing guidelines and supplying standardized patient instructions resulted in a significant reduction in opioid prescriptions.^22^ This serves as an important tool to facilitate the adoption of best practice recommendations as the development of clear-cut guidelines was faced with some objections due to concerns of over-restricting prescriptions in a highly variable patient population and procedures or restricting practice autonomy and amplifying legal strain.^2^

### Limitations and Future Recommendations

The main limitation is our reliance on patient memory in obtaining the telephone interview data which is not only subjective in nature but also at risk of recall bias. In an attempt to control for this risk of bias, we limited the study period to 3 months to avoid the effect of longer durations on patient recall. The cross-sectional design in the form of a telephone interview format did not allow us to obtain a long-term pain profile for each patient. A prospective design would be ideal for future studies. For health information protection, the data provided by the NSPMP program did not allow us to analyze the opioid prescriptions into daily equivalents for each patient. We recommend better tailoring of prescriptions for postoperative pain through mitigating opioid prescriptions to certain procedures, use of intraoperative nerve block, adding NSAIDs to the opioid regimen or multimodal pain therapy, and improving education regarding postoperative pain management, including expectations of pain and appropriate opioid pill use and disposal. Ideally, we aim to decrease the number of prescribed pills postoperatively without reducing patients’ care and comfort.

## Conclusion

In conclusion, our data showed that prescribing patterns vary considerably among patients undergoing common breast plastic surgeries and opioids are prescribed in excess for the breast procedures we analyzed. Furthermore, there was a substantial number of leftover opioid pills, which can be a source of diversion and abuse as the majority of unused opioids were noted to still be at home or disposed of inappropriately. The findings of this study suggest a role for reviewing opioid-prescribing patterns for common plastic surgery procedures to reduce the contribution to the ongoing opioid epidemic and serve as a primer to establish better opioid prescription guidelines in plastic surgery institutions.

## Supplemental Material

sj-docx-1-psg-10.1177_22925503231172789 - Supplemental material for Excess Opioid Medication and Variation in Prescribing Patterns Following Common Breast Plastic Surgeries Supplemental material, sj-docx-1-psg-10.1177_22925503231172789 for Excess Opioid Medication and Variation in Prescribing Patterns Following Common Breast Plastic Surgeries by Osama A. Samargandi, Colton Boudreau, Kaleigh MacIssac, Connor McGuire, Rawan ElAbd, Adel Helmi, and David Tang in Plastic Surgery
