# Supplementary material for: Excess Opioid Medication and Variation in Prescribing Patterns Following Common Breast Plastic Surgeries
Source: Plast Surg (Oakv). 2023 May 26;32(4):606–13. doi: 10.1177/22925503231172789 (PMC11489969; doi:10.1177/22925503231172789)
Supplement: sj-docx-1-psg-10.1177_22925503231172789 - Supplemental material for Excess Opioid Medication and Variation in Prescribing Patterns Following Common Breast Plastic Surgeries [file sj-docx-1-psg-10.1177_22925503231172789.docx]

**Telephone Script**

*Excess Opioid and Variation in Prescribing Patterns in Common Plastic Surgery Procedures*

*Romeo No. 1023725*

Hello, would Mr/Mrs/Ms (Insert full patient name) be in?

> Yes (proceed) / No (call back at a later time)

This is (researcher name) calling from the plastic surgery research team at the QE2 health science centre in Halifax. We are calling regarding your participation in a research study surrounding pain control following common plastic surgery procedures.

We are calling today because your surgery was approximately 6-8 weeks ago (State date of surgery to participant to confirm). We would like to ask you a series of questions. This shouldn’t take more than 3-5 minutes. Please be reminded that you are free to withdraw your participation if you would like.

Would you like to proceed?

>Yes (proceed)/No (thank them for their time and mark their file as withdrawn)

1. Do you know the name of the medication(s) you were prescribed following your surgery?
   1. Yes (list): ____________________________________________________
   2. Not sure (refer to medical records and ask participant if this is correct
   3. Cannot confirm
2. How would you rate your overall pain during the post-operative period, on a scale of 0-10, with 0 being no pain at all, and 10 being worst pain you have ever experienced?
   1. Rating (0-10) ____
3. How would you rate your pain SPECIFICALLY during the first 48-72 hours after your surgery? Once again, 0 being no pain at all, 10 being worst pain you’ve ever experienced.
   1. Rating (0-10) ____
4. Did you require a hospital stay following your surgery?
   1. Yes ___ - How long? ___ days / No ___
   2. If yes – How would you rate your pain during the period of your hospital stay? 0 being no pain at all, 10 being worst pain ever experienced?
      1. Rating (0-10) ____
5. Did you, at any point in your recovery, have to return to or contact your plastic surgeon or another physician regarding pain control?
   1. Yes (describe)__________________________________________
   2. No
6. Did you, at any point take ANY medications in addition to those prescribed to you by your plastic surgeon? For example, any over the counter medications?
   1. Yes ___
      1. What medications? ____________________________________
      2. How many/How often?__________________________________
   2. No ____

- Determine based on above information AND medical records/data link from NSPMP whether or not the patient was prescribed an opioid
  - If Yes – Proceed below / If no, conclude interview and thank participant (ask if any further questions and remind them that they can contact us at any time)

1. How many of the prescribed opioid pills did you take (number of tablets, if possible, or proportion ie. All, half, a few, etc.) __________________________________________
2. What did you do with any unused opioids? ___________________________________
3. Did you require any refills during your recovery period? _________________________

Thank you script/Debrief: We would like to thank you for your participation in our research study. We are very grateful for your time. Do you have any additional questions for us?

We would like to remind you that the end of this phone interview concludes your formal participation in the study. However, we are happy to be of assistance to you if you should have any questions regarding the study.

Thank you!
